# Tumor-derived exosomal lncRNA SNHG4 promotes triple-negative breast cancer progression by targeting XPO5

**DOI:** 10.3389/fonc.2025.1593827

**Published:** 2025-06-27

**Authors:** Zhi-Wen Wang, Hou-Sheng Yang, Hong-Shan Guo, Yue-Ying Li, Jin-Yun Zhong, Shu Jiang, Jia-Peng Li, Zhong-Yi Yang, Chuan-Yi Zhou, Jun Wang, Xing-Hua Liao, Lei Mao

**Affiliations:** ^1^ Institute of Biology and Medicine, College of Life and Health Sciences, Wuhan University of Science and Technology, Wuhan, China; ^2^ Key Laboratory of Chronic Noncommunicable Diseases, Yueyang Vocational Technical College, Yueyang, China; ^3^ School of Medicine, Hunan Normal University, Changsha, China; ^4^ Department of Gynecology and Obstetrics, Tianjin Key Laboratory of Female Reproductive Health and Eugenic, Tianjin Medical University General Hospital, Tianjin, China; ^5^ Department of Oncology, Yueyang People’s Hospital, Yueyang Hospital Afliated to Hunan Normal University, Yueyang, China

**Keywords:** TNBC, exosome, lncRNA SNHG4, XPO5, proliferation, migration

## Abstract

**Background:**

Triple-negative breast cancer (TNBC) is the subtype of advanced breast cancer with the shortest survival time and the poorest prognosis, and treatment options are relatively limited. Exosomes, small extracellular vesicles enriched with bioactive molecules, are critical mediators of intercellular communication and have been implicated in cancer progression. The aim of this study was to investigate the molecular mechanism of exosomes promoting the proliferation and migration of TNBC.

**Methods:**

In this study, exosomes were identified by Flow cytometry and transmission electron microscopy, and RNA sequencing (RNA-seq) was used to identify differentially expressed genes and downstream regulatory molecules in exosomes. RNA-seq results were supported by bioinformatics analysis and Western blot analysis. Functional assays including *in vivo* tumor formation, Colony formation Assay, Scratch migration and transwell assays were performed to study exosomes related phenomena and mechanism.

**Results:**

Serum-derived exosomes from patients with TNBC can induce TNBC progression *in vitro* and *in vivo*. lncRNA SNHG4 was most significantly up-regulated in exosomes, and overexpression of lncRNA SNHG4 significantly promoted the proliferation and migration of TNBC cells. In addition, lncRNA SNHG4 promotes TNBC cell proliferation and migration by upregulating the expression of Exportin 5(XPO5). Silencing XPO5 can effectively attenuate the tumor-promoting effect of serum exosomes in TNBC patients. Mechanistically, lncRNA SNHG4 acts through XPO5-mediated pathways. Silencing XPO5 can effectively inhibit the tumor-promoting effect mediated by lncRNA SNHG4.

**Conclusions:**

Taken together, our study revealed that the exosome lncRNA SNHG4 exerts its oncogenic role by activating XPO5-mediated pathways, thereby regulating TNBC cell proliferation and migration. This can be considered as a potential target for TNBC molecular therapy.

## Introduction

Triple-negative breast cancer (TNBC) accounts for about 15% -20% of all breast cancers and is the deadliest breast cancer subtype characterized by a lack of oestrogen receptor (ER), progesterone receptor (PR) and human epidermal growth factor receptor 2 (HER-2) expression (1, 2). At present, there is still a lack of effective treatment for TNBC, and chemotherapy is still the main treatment (3). However, TNBC is highly heterogeneous and aggressive, and conventional anthracycline/taxane-based postoperative adjuvant chemoradiotherapy often does not completely eliminate the primary tumor and residual metastatic lesions; It eventually leads to 40% tumor recurrence, treatment failure and death (4, 5). Therefore, there is an urgent need for a deeper understanding of the early diagnosis and molecular mechanisms underlying TNBC to identify effective biomarkers and therapeutic targets.

Exosomes are small extracellular vesicles with diameters ranging from 30 to 150 nm, derived from endosomes and released by cells. They are widely present in various body fluids such as blood, saliva, and urine, carrying a variety of biomolecules, including proteins, lipids, and nucleic acids (such as long non-coding RNAs (lncRNAs)), which facilitate intercellular communication (6, 7). Exosomes not only play a crucial role in cellular communication but also contribute to a variety of physiological and pathological processes, particularly in cancer development, progression, and metastasis (8). Exosomes secreted by cancer cells can transfer specific bioactive molecules to receptor cells, thereby influencing the tumor microenvironment (TME) and promoting tumor growth, metastasis, and drug resistance (9). Moreover, the biomolecules within exosomes, particularly RNA molecules, are considered potential tools for monitoring disease progression and treatment responses (10). In TNBC, exosomes from patient have been shown to modulate the TME and promote the growth and metastasis of tumor cells (11, 12). However, studies on the molecular composition of exosomes in the serum of TNBC patients and their specific role in TNBC progression are limited, particularly the role of exosomal lncRNAs in tumor development.

Non-coding RNAs (ncRNAs) refer to RNA molecules that do not encode proteins. Their roles in gene expression regulation, cellular functions, and cancer progression have garnered increasing attention in recent years (13). LncRNAs are a class of ncRNAs that are typically longer than 200 nucleotides, and they have been shown to play diverse roles in regulating gene expression, cell differentiation, and cancer cell invasion and metastasis (13, 14). LncRNA small nucleolar RNA host gene 4 (SNHG4) is a specific long non-coding RNA that belongs to the SNHG family (15). Recent studies have shown that SNHG4 is highly expressed in various cancers and is involved in regulating multiple cancer-related biological processes, making it closely associated with clinical pathological features and prognosis in some cancers (16–18). However, the study of lncRNA SNHG4 in TNBC remains limited, particularly regarding its role in TNBC patient serum exosomes and its impact on tumor progression.

In this study, we explored the expression pattern of lncRNA SNHG4 in exosomes derived from the serum of TNBC patients and evaluated whether TNBC-derived exosomes could induce tumor progression *in vitro* and *in vivo*. Our findings indicate that lncRNA SNHG4 is significantly overexpressed in TNBC serum exosomes and promotes TNBC cell proliferation and migration via Exportin 5 (XPO5). Inhibition of XPO5 effectively weakens the pro-tumor effects of TNBC patient serum exosomes. We propose that lncRNA SNHG4 and XPO5 may serve as novel therapeutic targets for TNBC. The results of this study could provide new insights into early diagnosis and treatment strategies for TNBC.

## Materials and methods

### Cell culture

The human TNBC cell lines, MDA-MB-231 and BT549, were cultured in DMEM (Gibco, USA), respectively. The culture media were supplemented with 10% fetal bovine serum (FBS, Vazyme, China) and 1% penicillin-streptomycin (Servicebio, China). All cells were maintained in a humidified incubator at 37°C with 5% CO_2_. The stable overexpression of lncRNA SNHG4 cells were achieved by infecting cells with lentivirus carrying the SNHG4 sequence and a puromycin resistance gene, followed by puromycin selection and single-clone isolation. The lentiviral vectors were produced by GeneChem (Shanghai GeneChem Co., Ltd., China), while the construction and validation were performed in our laboratory.

### Clinical tissue sample collection

Clinical samples were collected from the Wuhan University of Science and Technology Hospital of Science and Technology, between January 2022 and June 2023. Blood samples were collected from 30 patients with pathologically confirmed TNBC patients, and serum was separated by centrifugation at 2000 g for 10 minutes at 4°C after clotting. The resulting supernatant was stored at -80°C until further use. All patients did not receive chemotherapy or radiotherapy before surgery, and signed a written informed consent. The study was approved by the Ethics Committee of Wuhan University of Science and Technology, following the Declaration of Helsinki.

### Preparation of exosomes

Serum samples were mixed with PBS in a 1:1 ratio. After filtration through a 0.22 μm filter, the serum underwent a series of centrifugations: the first at 300 g for 10 minutes at 4°C, the second at 2,000 g for 10 minutes at 4°C, and the third at 10,000g for 60 minutes at 4°C. To isolate the exosomes, ultracentrifugation was performed using a Beckman ultracentrifuge (Beckman Coulter, USA) with an SW 32 Ti rotor at 100,000 g for 90 minutes at 4°C. The pellet was resuspended in PBS, and the process was repeated. The final pellet was aliquoted and stored at -80°C.

### Transmission electron microscopy assay

For TEM imaging, exosome samples were negatively stained with 1% phosphotungstic acid (Aladdin, China). Samples were examined using a Hitachi HA7100 transmission electron microscope (Hitachi, Japan) at an operating voltage of 80 kV.

### Nano-flow cytometry assay

The particle size and concentration of exosomes from TNBC patient serum were analyzed using a flow nanoanalyzer (nanoFCM, China). The S23M-SEV (nanoFCM, China) was used as a particle size standard, and the Quality Control Nanospheres Series (nanoFCM, China) served as the concentration standard. Data were processed using the NF Profession 1.16 software.

### Flow cytometry assay

Flow cytometric analysis was performed to detect exosomal markers. Exosomes were stained with CD63 (BD, USA, 550759) and CD81 (BD, USA, 551108) antibodies according to the manufacturer’s instructions. Flow cytometry was conducted using a SONY ID7000 flow cytometer (Sony, Japan).

### Cellular uptake assay

Exosomes were labeled with PKH26 (Sigma-Aldrich, USA) according to the manufacturer’s protocol. Briefly, 5 μL of exosome solution and 5 μL of PKH26 dye were mixed with Diluent C to a final volume of 100 μL and incubated for 10 minutes. Excess dye was removed using an Exosome Spin Column (Sigma-Aldrich, USA). Labeled exosomes were then incubated with target cells for 1 hour. Cellular uptake was assessed using an Olympus confocal microscope (Olympus, Japan).

### Western blot

Protein extraction was performed using RIPA lysis buffer (Servicebio, China), and protein concentrations were determined using a BCA Protein Assay Kit (Servicebio, China). Equal amounts of protein were separated by SDS-PAGE (Servicebio, China) and transferred onto polyvinylidene fluoride (PVDF) membranes (Merck Millipore, Germany). Membranes were blocked using Rapid Blocking Buffer (Servicebio, China) and incubated with primary antibodies targeting XPO5 (Abclonal, China, A6790), CD63 (Abclonal, China, A5271), ALIX (Abclonal, China, A2215), TSG101 (Abclonal, China, A5789), Calnexin (Abclonal, China, A24433), or GAPDH (Abclonal, China, A19054). Following primary antibody incubation, membranes were probed with HRP-conjugated secondary antibody (Abclonal, China, AS014). Protein bands were visualized using enhanced chemiluminescence (ECL) reagents (Meilunbio, China) and imaged with a ChemiDoc imaging system (Bio-Rad, USA).

### Quantitative real-time PCR

Total RNA was extracted from cells using the FastPure Cell/Tissue Total RNA Isolation Kit V2 (Vazyme, China). cDNA synthesis for lncRNAs was performed using the lnRcute lncRNA cDNA First Strand Synthesis Kit (without genomic DNA remover) (Tiangen, China), while mRNA reverse transcription was conducted using the HiScript III 1st Strand cDNA Synthesis Kit (+gDNA wiper) (Vazyme, China). qRT-PCR was performed on a Bio-Rad CFX system (Bio-Rad, USA) with SYBR Green Master Mix (Vazyme, China). Primers for qRT-PCR were designed and synthesized by Shanghai Shenggong Biotechnology Co., Ltd. The sequence of gene-related primers is as follows:

GAPDH-Forward primer: GTCTCCTCTGACTTCAACAGCG, GAPDH-Reverse primer: ACCACCCTGTTGCTGTAGCCAA; XPO5-Forward primer: GCAAGGAGTCTGACCTGTTG, XPO5-Reverse primer: CCTTCTCCAGGTTTCCAGGT; lncRNA SNHG14-Forward primer: GCAGATGCAACTGCAGGATGAC, lncRNA SNHG14-Reverse primer: TGTCACTTCAGGTGAAGCATCTG.

### Lentiviral transfection and stable cell line construction

Lentiviral packing plasmids VSVG and Gag were co-transfected into HEK 293T cells with target plasmids using Lipo3000™ transfection reagents. HEK 293 T cells were used to produce lentivirus. After TNBC cells were seeded in dishes or plates and cultured overnight, lentivirus and target cells were co-cultured for 72 h. After 72 h, transfected cells were selected with 2 μg/mL puromycin for 1–2 weeks depending on antibiotic marker.

### Animal model

Nude mouse (4–6 weeks old, female) were purchased from Jicui Yikang Biotechnology Co., Ltd. (China). Mouse were subcutaneously injected with MDA-MB-231 (2×10 (7) cells/100 μL/mouse). All procedures were performed in accordance with the ethical guidelines established by the Animal Care and Use Committee of Wuhan University of Science and Technology. Tumor growth was monitored through caliper measurements, and body weight was recorded. When tumors reached a volume of 2000 mm³ or when mouse exhibited signs of distress, euthanasia was performed according to ethical guidelines. Tumors were subsequently collected for further analysis.

### Immunohistochemistry

Formalin-fixed, paraffin-embedded tumor tissues were sectioned and stained with anti-XPO5 antibody (Abclonal, China, A6790) according to the standard IHC protocol.

### CCK-8 assay

Cell viability was assessed using the Cell Counting Kit-8 (CCK-8, Vazyme, China). Briefly, cells were plated in 96-well plates and subjected to the specified treatment conditions. At designated time points (24 hours, 48 hours, 72 hours), CCK-8 solution (diluted with serum-free medium) was added to each well and incubated for 1–4 hours at 37°C in a 5% CO_2_ incubator. Absorbance at 450 nm was measured using a microplate reader (BioTek Instruments, USA). Each experimental condition was performed in triplicate, with wells containing only CCK-8 solution serving as the blank control.

### Colony formation assay

For the colony formation assay, cells were seeded at a low density in 6-well plates and cultured for 14 days. Colonies were fixed with methanol and stained with 0.1% crystal violet (Beyotime Biotechnology, China). Colonies were manually counted under an Olympus light microscope (Olympus Corporation, Japan).

### Wound scratch assay

To assess cell migration, a confluent monolayer of cells in 6-well plates was scratched with a sterile pipette tip to create a wound. Detached cells were removed by washing with PBS. The migration of cells into the wound area was monitored at 0 and 48 hours using an Olympus phase-contrast microscope (Olympus Corporation, Japan). Wound closure was quantified by measuring the area between the edges of the wound.

### Transwell assays

Cell migration were assessed using Corning Transwell chambers (8.0 µm pore size). Cells were seeded into the upper chamber in serum-free medium, while the lower chamber contained medium supplemented with 10% FBS as a chemoattractant. After 48 hours, cells that migrated or invaded through the membrane were fixed with methanol and stained with 0.1% crystal violet (Beyotime Biotechnology, China). The number of cells was quantified using a microscope.

### RNA sequencing and bioinformatics analysis

Total RNA was extracted from cells and stored at -80°C until further analysis. Transcriptome sequencing was performed by Beijing Qingke Biotechnology Co., Ltd. Bioinformatic analyses were conducted using R software and various bioinformatics packages.

### Statistical analysis

Data are expressed as mean ± standard deviation (SD) from at three independent experiments. Statistical significance between groups was assessed using Student’s *t*-test, one-way ANOVA, or two-way ANOVA followed by *post-hoc* tests where applicable. A *p*-value < 0.05 was considered statistically significant. All statistical analyses were performed using GraphPad Prism software (GraphPad Software, USA).

## Result

### Extracellular vesicle isolation from TNBC patient serum

**Figure 1:**
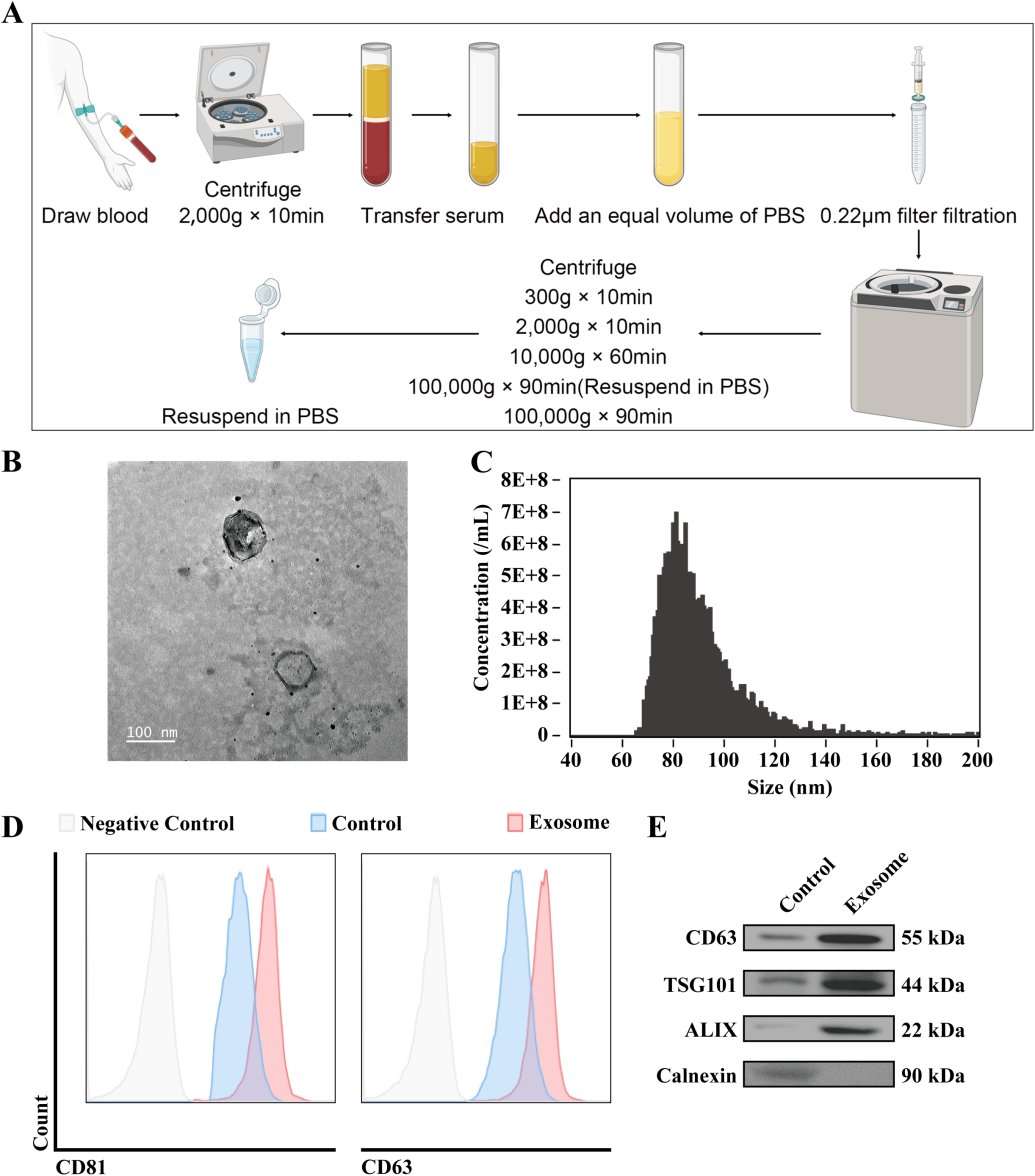
Extracellular vesicle isolation from TNBC patient serum. **(A)** Schematic representation of the protocol for the isolation of exosomes from TNBC patient serum. **(B)** Representative TEM images of isolated exosomes. The accelerating voltage was set to 80 kV. Scale bar, 100 μm. **(C)** Nanoflow was used to determine the size distribution of the isolated exosomes. **(D)** Flow cytometry analysis was performed to detect the membrane expression of exosomal markers CD63 and CD81. **(E)** WB analysis was conducted to assess the expression of exosomal markers CD63, TSG101, ALIX and Calnexin. Control: Supernatant after the first centrifugation at high speed.

Exosomes were isolated from the serum of 30 TNBC patients using differential ultracentrifugation, as outlined in the materials and methods section. This process yielded a pellet enriched in extracellular vesicles (**Figure 1A**). Transmission electron microscopy (TEM) was utilized to examine the morphology of the isolated vesicles, revealing a characteristic double-layered membrane structure typical of exosomes (**Figure 1B**). Nanoparticle tracking analysis (NTA) of the exosome suspension showed a size distribution with a peak diameter ranging from 70–130 nm, and a mean size of 90.7 ± 18.8 nm (**Figure 1C**), which is consistent with the size range typically associated with exosomes. Flow cytometry analysis further confirmed the exosomal nature of the vesicles, detecting the expression of exosomal markers CD63 and CD81, both of which showed positive expression (**Figure 1D**). The results show that compared to the supernatant after the initial centrifugation, CD63, TSG101, and ALIX exhibited positive signals in the proteins after the second centrifugation, while calreticulin showed negative results, in accordance with the MISEV exosome characterization guidelines (**Figure 1E**). These findings collectively confirm the successful isolation and characterization of exosomes from the serum of TNBC patients, providing a foundation for further molecular and functional investigations.

### Promotion of tumor proliferation and migration by TNBC patient-derived serum exosomes

**Figure 2:**
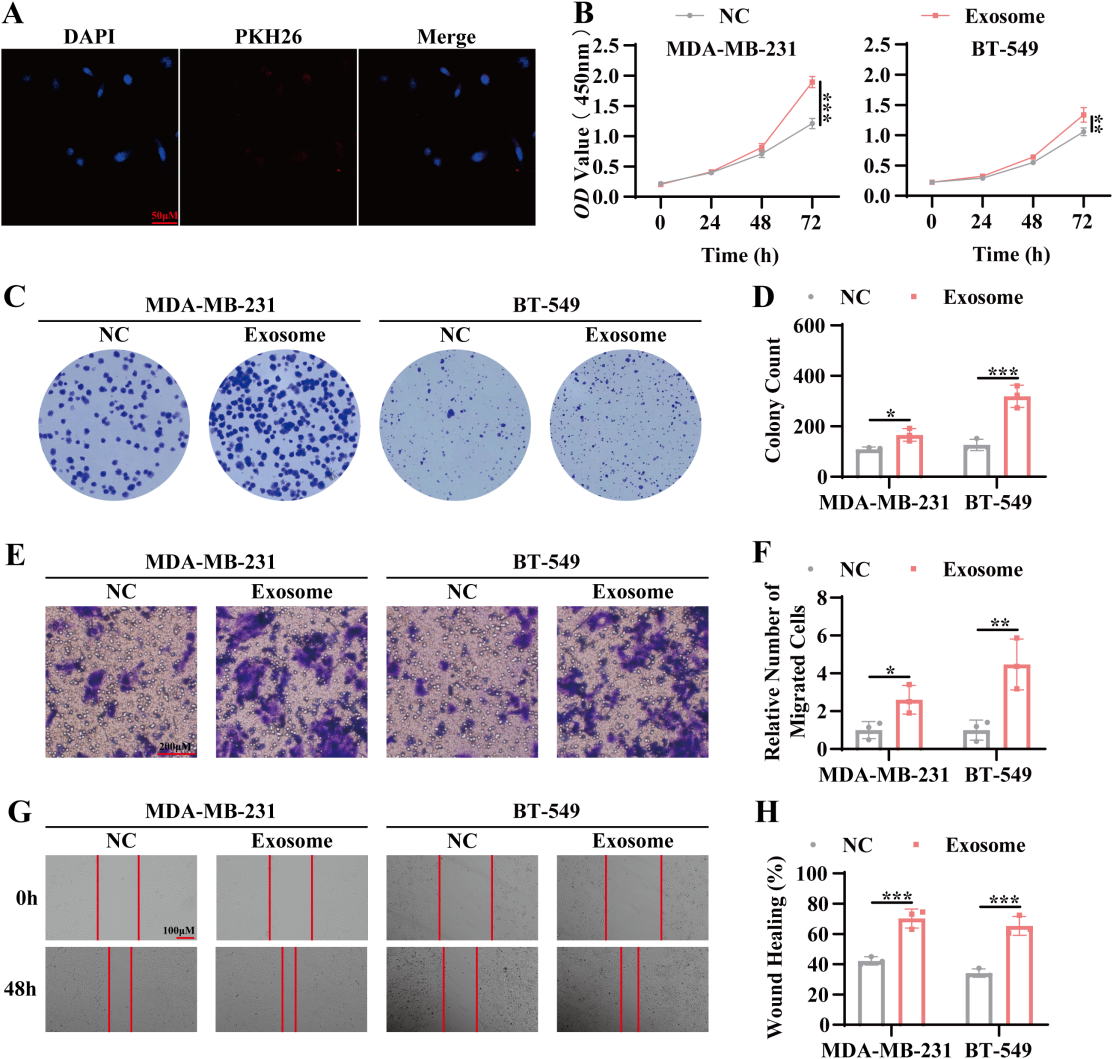
Promotion of tumor proliferation and migration by TNBC patient-derived serum exosomes. **(A)** Confocal microscopy was used to examine the uptake of exosomes by tumor cells. Blue: DAPI (nuclear staining); Red: PKH26 (exosome labeling). Scale bar, 100 μm. **(B)** CCK-8 assay was performed to assess cell viability following co-culture of tumor cells with exosomes for 24 hours, 48 hours, and 72 hours. **(C)** Colony formation assay was conducted to evaluate the proliferative capacity of tumor cells following 7 days of co-culture with exosomes. **(D)** Statistical analysis of the colony formation assay. **(E)** Transwell migration assay was used to measure the migratory capacity of tumor cells co-cultured with exosomes for 72 hours. Scale bar, 100 μm. **(F)** Quantitative analysis of the transwell migration assay. **(G)** Scratch wound healing assay was performed to evaluate the migratory ability of tumor cells co-cultured with exosomes for 72 hours. Scale bar, 100 μm. **(H)** Statistical analysis of the scratch wound healing assay. Data are presented as Mean ± SD. Statistical significance was determined as follows: *p < 0.05, **p < 0.01, ***p < 0.001, *ns* > 0.05.

To evaluate the effects of exosomes derived from the serum of TNBC patients on tumor cell behavior, co-culture experiments were conducted. Initial uptake assays confirmed the internalization of the isolated exosomes by tumor cells (**Figure 2A**). CCK8 and colony formation assays revealed a marked increase in the proliferative capacity of tumor cells following co-culture with TNBC patient-derived serum exosomes, evidenced by a higher number of colonies and increased optical density (*OD*) values (**Figures 2B–D**). Furthermore, Transwell migration and scratch wound assays demonstrated enhanced migratory activity in tumor cells exposed to TNBC patient-derived exosomes, with a significant increase in cell movement compared to controls (**Figures 2E–H**). These results suggest that exosomes from TNBC patient serum facilitate both tumor cell proliferation and migration, highlighting their potential role in TNBC pathogenesis.

### Exosome-mediated tumor progression in a nude mouse xenograft model

**Figure 3:**
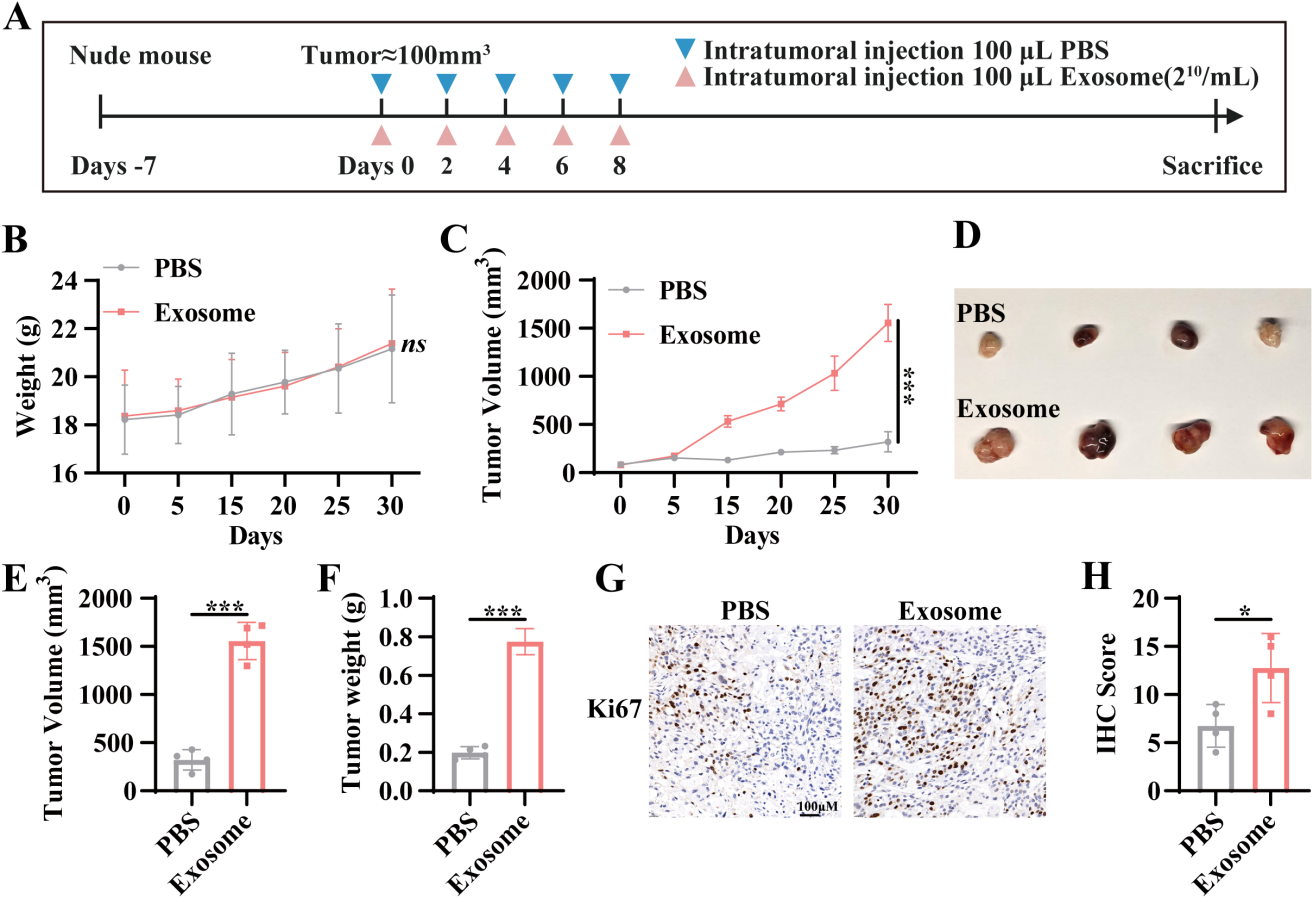
Exosome-mediated tumor progression in a nude mouse xenograft model. **(A)** Schematic representation of the intervention protocol in the nude mouse xenograft model. **(B)** Body weight curve of nude mouse during the experiment. **(C)** Tumor volume growth curve in nude mouse following intervention. **(D)** Subcutaneous tumors were excised at the experimental endpoint. **(E)** Measurement of tumor volume. **(F)** Measurement of tumor mass. **(G)** Immunohistochemical **(IHC)** analysis was performed to assess Ki67 expression levels in tumors from different experimental groups. **(H)** Quantitative analysis of IHC staining for Ki67. Tumor volume was calculated as length × width × width/2. Data are presented as Mean ± SD. Statistical significance was determined as follows: *p < 0.05, **p < 0.01, ***p < 0.001, *ns* > 0.05.

To investigate the *in vivo* effects of exosomes isolated from TNBC patient serum on tumor progression, a nude mouse xenograft model was employed. As outlined in the experimental design, TNBC patient-derived serum exosomes were administered via intratumoral injection (**Figure 3A**). No significant changes in the body weight of the mouse were observed throughout the treatment period (**Figure 3B**), indicating the absence of systemic toxicity following exosome administration. However, tumors in the exosome-treated group demonstrated accelerated growth compared to the control group (**Figure 3C**). Upon euthanasia, tumor volume and mass were significantly greater in the exosome-treated group than in the control group, as evidenced by measurements of the excised subcutaneous tumors (**Figures 3D–F**). Immunohistochemical analysis of the excised tumors revealed significantly higher Ki67 expression in the exosome intervention group, further confirming enhanced tumor proliferation (**Figures 3G, H**). These results indicate that exosomes derived from TNBC patient serum promote tumor progression *in vivo* by enhancing tumor growth and proliferation.

### Differential gene expression and functional enrichment analysis in TNBC-derived serum exosomes

**Figure 4:**
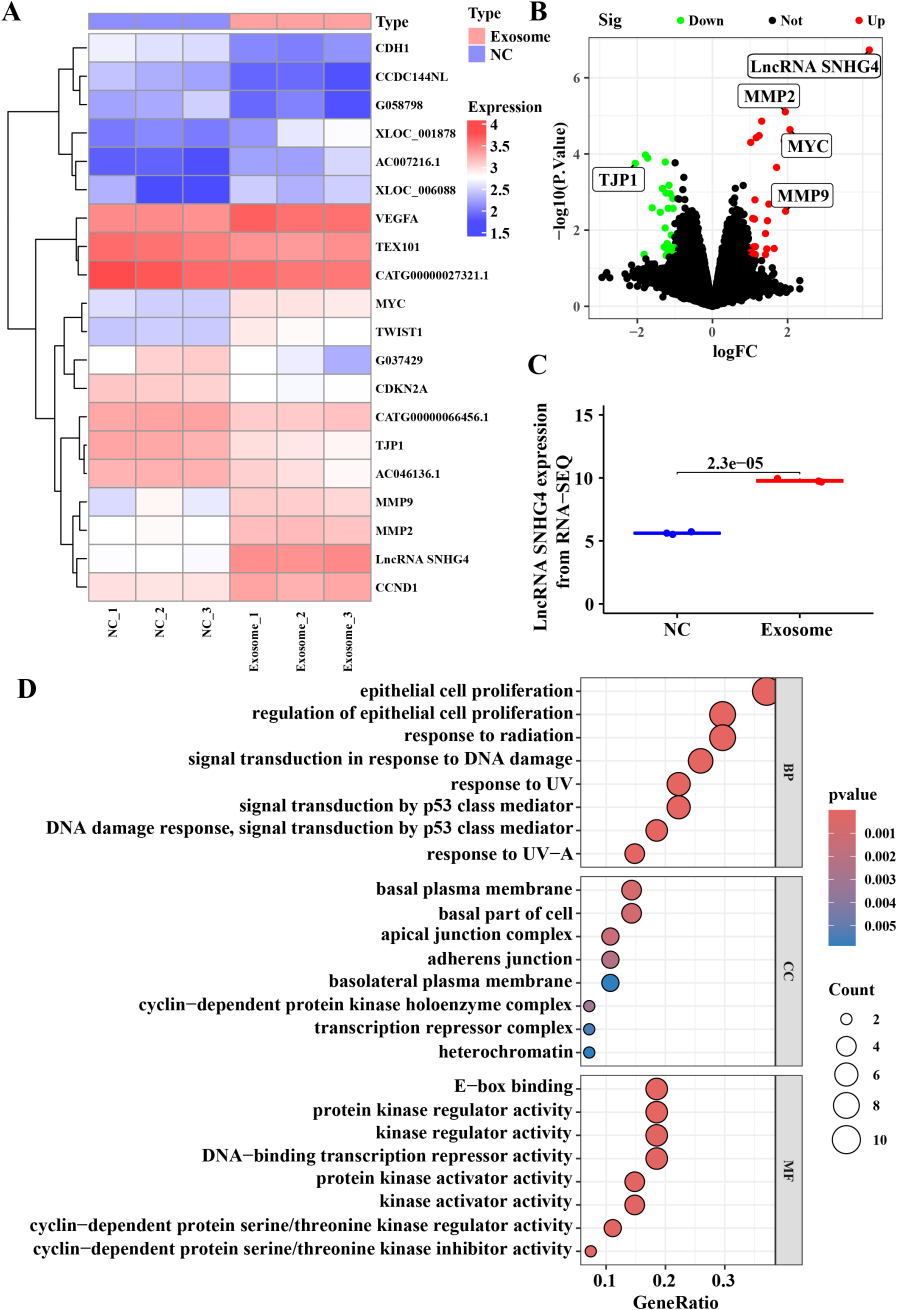
Differential gene expression and functional enrichment analysis in TNBC-derived serum exosomes. **(A)** Heatmap of differentially expressed genes based on RNA sequencing analysis. Differentially expressed genes were identified using the limma package with a log fold change >2 and *p*-value <0.05. **(B)** Volcano plot representing the distribution of differentially expressed genes, with upregulated genes shown in red and downregulated genes in green. **(C)** Relative expression levels of lncRNA SNHG4 in RNA sequencing data. **(D)** Gene ontology (GO) functional enrichment analysis for biological processes.

To further elucidate the molecular mechanisms underlying the effects of TNBC patient-derived exosomes on tumor progression, RNA sequencing was performed on the isolated exosomes. Heatmap and volcano plot analyses identified 61 differentially expressed genes with a log-fold change greater than 2 and a *p*-value less than 0.05, of which 27 were upregulated and 34 were downregulated (**Figures 4A, B**). The long non-coding RNA SNHG4 was found to be the most significantly altered gene. Quantification of SNHG4 expression confirmed a significant increase in exosomes derived from TNBC patient serum (**Figure 4C**). Bioinformatics functional enrichment analysis revealed that multiple pathways associated with cell proliferation and migration were significantly enriched, including the regulation of epithelial cell proliferation (**Figure 4D**). KEGG and Reactome pathway enrichment analyses further identified pathways related to cell adhesion and migration, such as Adherens junction signaling and Relaxin signaling (**Supplementary Figure S1A**). Additionally, pathways involved in the Mitotic G1 phase, G1/S transition, and Cyclin D-associated events in G1 were also significantly enriched (**Supplementary Figure S1B**), supporting the conclusion that exosomes from TNBC patient serum promote tumor progression by modulating proliferative and migratory processes in tumor cells.

### Overexpression of lncRNA SNHG4 enhances tumor proliferation and migration

**Figure 5:**
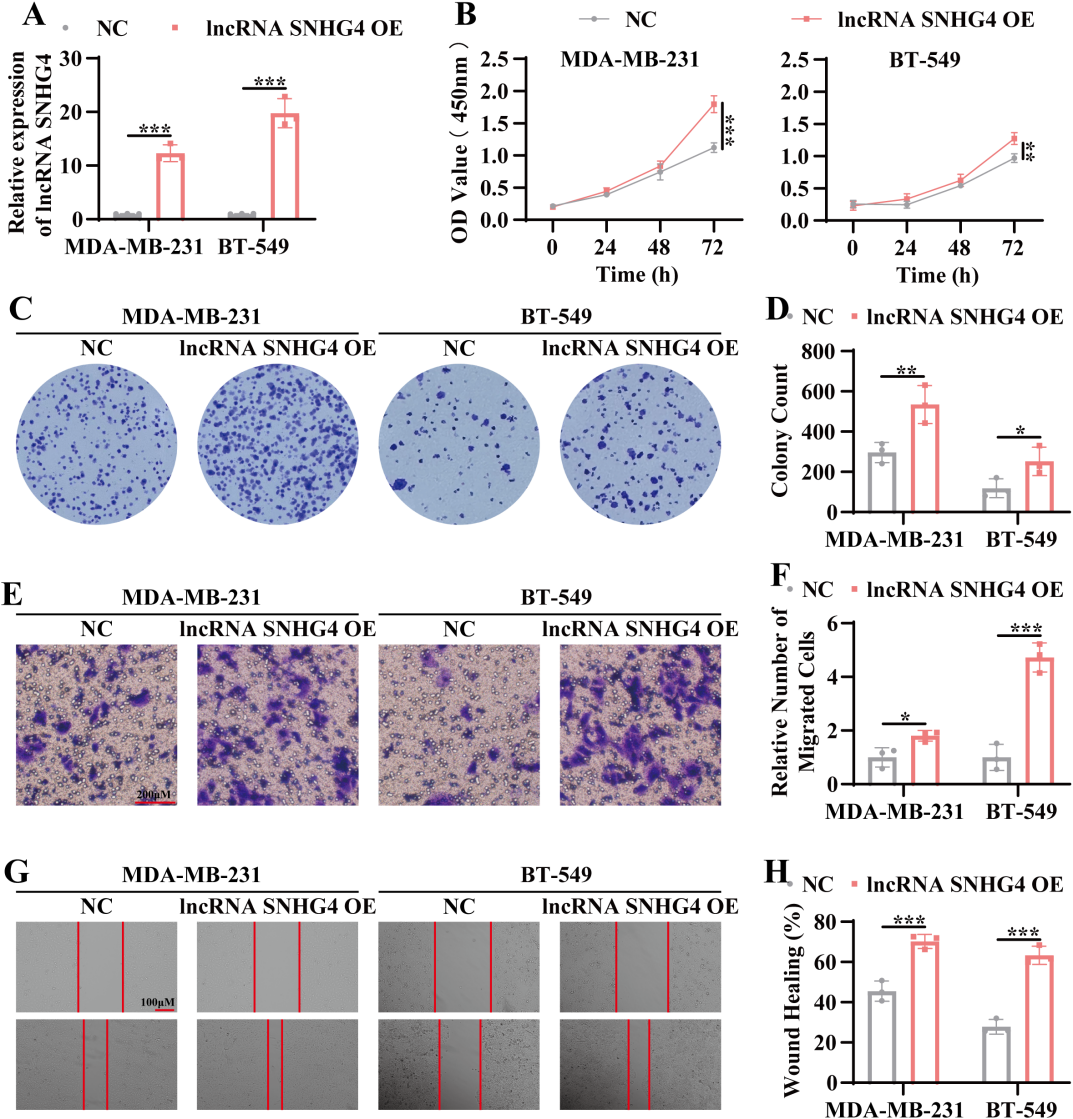
Overexpression of lncRNA SNHG4 enhances tumor proliferation and migration. **(A)** QRT-PCR analysis to assess the relative expression levels of lncRNA SNHG4 in stable overexpression cell lines of lncRNA SNHG4 and wild-type TNBC cell lines. **(B)** CCK-8 assay to measure cell viability in stable lncRNA SNHG4-overexpressing cell lines and wild-type cell lines at 24 hours, 48 hours, and 72 hours post-culture. **(C)** Colony formation assay to evaluate the *in vitro* clonogenic potential of lncRNA SNHG4-overexpressing cell lines compared to wild-type cell lines. **(D)** Statistical analysis of colony formation assay data. **(E)** Transwell migration assay to assess the *in vitro* migratory ability of lncRNA SNHG4-overexpressing cell lines and wild-type cell lines. Scale bar, 100 μm. **(F)** Quantitative analysis of transwell migration assay data. **(G)** Scratch wound healing assay to evaluate the migratory capacity of lncRNA SNHG4-overexpressing cell lines and wild-type cell lines. Scale bar, 100 μm. **(H)** Statistical analysis of scratch wound healing assay results. Data are presented as Mean ± SD. Statistical significance was determined as follows: *p < 0.05, **p < 0.01, ***p < 0.001, *ns* > 0.05.

Based on the RNA sequencing analysis, which identified lncRNA SNHG4 as a potential regulator of TNBC progression, we sought to determine whether overexpression of SNHG4 could promote tumor progression. A stable lncRNA SNHG4-overexpressing TNBC cell line was generated using a lentiviral expression system. As shown in **Figure 5A**, lncRNA SNHG4 expression was significantly increased in both MDA-MB-231 and BT549 cell lines. Subsequent functional assays, including CCK8 and colony formation assays, demonstrated a marked increase in cell proliferation in the lncRNA SNHG4-overexpressing lines compared to control cells (**Figures 5B–D**). Moreover, migratory capacity was assessed using Transwell and wound healing assays, revealing a significant enhancement in migration in the SNHG4-overexpressing cells (**Figures 5E–H**). These results align with the findings observed following exosome treatment, suggesting that overexpression of lncRNA SNHG4 promotes both proliferation and migration in TNBC cells.

### Exosome-mediated tumor progression through lncRNA SNHG4 and XPO5 regulation

**Figure 6:**
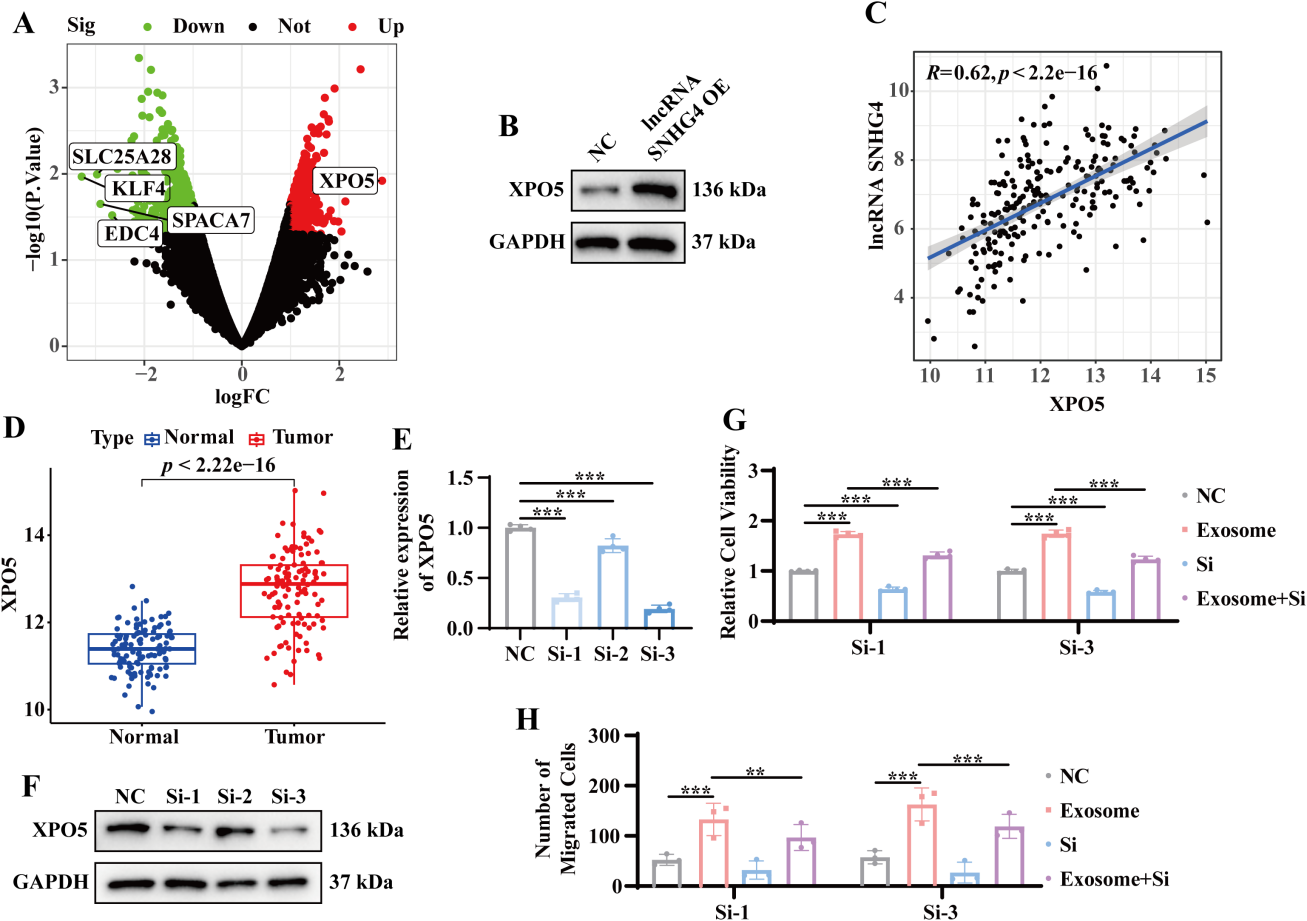
Exosome-mediated tumor progression through lncRNA SNHG4 and XPO5 regulation. **(A)** Volcano plot of differential gene expression in stable lncRNA SNHG4-overexpressing versus wild-type MDA-MB-231 cells based on RNA sequencing. **(B)** WB analysis to detect XPO5 protein expression in stable lncRNA SNHG4-overexpressing and wild-type MDA-MB-231 cells. **(C)** Correlation analysis of lncRNA SNHG4 and XPO5 expression in TNBC samples from the TCGA database using the Starbase platform. **(D)** Expression levels of XPO5 in tumor and normal tissues from TNBC samples in the TCGA database. **(E)** QRT-PCR analysis to detect relative XPO5 expression levels in MDA-MB-231 cells 48 hours after transfection with XPO5 siRNA. **(F)** WB analysis of XPO5 expression in MDA-MB-231 cells 72 hours after transfection with XPO5 siRNA. **(G)** CCK-8 assay to assess relative cell viability following co-culture of tumor cells with exosomes or transfection with siRNA, or combined treatment for 72 hours. **(H)** Transwell migration assay to evaluate relative cell migration ability in MDA-MB-231 cells co-cultured with exosomes or transfected with siRNA, or combined treatment for 72 hours. Data are presented as Mean ± SD. Statistical significance was determined as follows: *p < 0.05, **p < 0.01, ***p < 0.001, *ns* > 0.05.

Previous results indicated that exosomes derived from the serum of TNBC patients significantly upregulate lncRNA SNHG4, which in turn enhances tumor cell proliferation and migration. To further investigate the molecular mechanisms underlying this effect, RNA sequencing was performed on stable lncRNA SNHG4-overexpressing and wild-type MDA-MB-231 cell lines. The top five most differentially expressed protein-coding genes were identified as SLC25A28, KLF4, EDC4, SPACA7, and XPO5 (**Figure 6A**). Notably, XPO5 protein expression was significantly elevated in the lncRNA SNHG4-overexpressing MDA-MB-231 cells (**Figure 6B**). Correlation analysis of TCGA TNBC data revealed a positive correlation between the expression levels of lncRNA SNHG4 and XPO5 (r = 0.62, *p* < 0.05) (**Figure 6C**). Additionally, compared with the adjacent tissues, the expression level of XPO5 was increased in TNBC tumor tissues (**Figure 6D**), suggesting that XPO5 high expression may contribute to TNBC progression. To further investigate the role of XPO5, siRNA-mediated knockdown of XPO5 was performed in both stable lncRNA SNHG4-overexpressing and wild-type MDA-MB-231 cells. The results show that knocking down XPO5 does not affect the expression of lncRNA SNHG4 (**Supplementary Figure S2A**). CCK8 and Transwell assays revealed that in cells with overexpression of lncRNA SNHG4, knocking down XPO5 weakened the enhanced proliferation and migration abilities (**Supplementary Figure S2B-C**). These results indicate that lncRNA SNHG4 regulates tumor cell proliferation and migration through modulation of XPO5 expression. To confirm that exosomes from TNBC patient serum also promote proliferation and migration via this pathway, similar experiments were performed in MDA-MB-231 cells exposed to exosomes or transfected with siRNA. As expected, knockdown of XPO5 in these cells diminished the ability of exosomes to promote proliferation and migration (**Figures 6E–H**). These findings collectively suggest that exosomes derived from TNBC patient serum mediate tumor progression through the lncRNA SNHG4-XPO5 axis.

## Discussion

TNBC is the leading cause of death from breast cancer in women, accounting for 5% of cancer-related deaths each year (19, 20). Although the existing treatment including surgery, radiotherapy and chemotherapy, but the results are still limited (21). In particular, TNBC patients exhibit high recurrence rates and short survival periods after receiving traditional treatments; especially in cases of tumor metastasis, the survival time may be shortened (22). Therefore, there is an urgent need for innovative therapeutic strategies to improve the accuracy of early diagnosis and enhance treatment efficacy.

In the current challenging clinical landscape of TNBC, exosomes have increasingly attracted attention due to their pivotal role in tumor progression as important mediators of intercellular communication. Exosomes were originally thought to be cell waste. However, recent studies have demonstrated that they play crucial roles in intercellular signal transmission, particularly in cancer development and metastasis (23, 24). Within the TME, exosomes influence tumor cell behavior and interactions with surrounding cells by carrying specific proteins, lipids, and nucleic acids. These vesicles not only promote tumor cell proliferation and migration but also contribute to immune evasion and the development of drug resistance (25–27). Research on TNBC-related exosomes has revealed that exosomal EDIL3 serves as a promising diagnostic biomarker for the early detection of TNBC (28). Beyond early diagnosis, exosomes are also involved in regulating TNBC biological functions. FOXM1 from exosomes derived from TNBC can promote cancer progression by activating IDO1 transcription in macrophages to inhibit ferroptosis and induce M2 polarization in tumor-associated macrophages (29). Consistent with these findings, our study discovered that exosomes derived from the serum of TNBC patients significantly promoted the proliferation and migration of TNBC cells. Additionally, in a nude mouse model, intratumoral injection of these exosomes further confirmed their ability to accelerate subcutaneous xenograft tumor growth. These results underscore the significant role of TNBC-derived exosomes in the TME and provide a theoretical foundation for future exosome-based therapeutic strategies.

Furthermore, RNA-seq analysis identified lncRNA SNHG4 as the most significantly altered transcript in serum exosomes derived from TNBC patients. This finding suggests that lncRNA SNHG4 may play a pivotal role in the molecular mechanisms mediated by serum exosomes in TNBC. Human RNA is classified into coding RNAs and non-coding RNAs (ncRNAs). Notably, over 98% of the human transcriptome comprises ncRNAs (30, 31). NcRNAs include microRNAs (miRNAs), long non-coding RNAs (lncRNAs), circular RNAs (circRNAs), among others. Among these, lncRNAs have attracted significant research interest due to their diverse roles in gene regulation (32). LncRNAs can modulate gene expression through various mechanisms, such as acting as competing endogenous RNAs (ceRNAs), influencing chromatin remodeling, and regulating transcription and translation (14, 33). They are involved in the regulation of multiple biological processes, including tumorigenesis and cancer progression. LncRNA SNHG4, a member of the small nucleolar RNA host gene (SNHG) family, has been identified as dysregulated in various cancers (34). Aberrant expression of lncRNA SNHG4 has been reported in gastric cancer (16), neuroblastoma (18), suggesting its potential role in tumor development and progression. However, the effect of lncRNA SNHG4 on TNBC progression remains unclear. To investigate the role of lncRNA SNHG4 in the pathogenesis of TNBC. In the present study, we adopted lentiviral transduction to overexpress lncRNA SNHG4 in TNBC cells, and the results showed that increased lncRNA SNHG4 expression significantly promoted cell proliferation and migration in TNBC. This finding suggests that lncRNA SNHG4 may be a potential therapeutic target for TNBC.

Subsequently, we identified the gene XPO5, which exhibits a high positive correlation with lncRNA SNHG4. XPO5 is a crucial nuclear export protein primarily responsible for transporting precursor microRNAs (pre-miRNAs) from the nucleus to the cytoplasm for further processing into mature miRNAs (35). An increasing number of cancer studies have demonstrated that XPO5 possesses oncogenic properties. XPO5 is upregulated in various cancer types, including colorectal cancer (CRC) and prostate adenocarcinoma (PRAD), and its overexpression is associated with unfavorable clinicopathological features and poor patient prognosis (36, 37). For instance, in CRC cells, inhibition of XPO5 expression results in reduced cell proliferation and invasiveness, and induces G1-S phase cell cycle arrest, indicating that XPO5 plays a pivotal role in maintaining cancer cell growth and metastatic potential (37). Our study shows that XPO5 expression is significantly elevated in tumor tissues compared with normal tissues. Patients with high expression of XPO5 had a lower survival rate. Inhibition of XPO5 expression can effectively inhibit the proliferation and migration of TNBC cells. This finding further supports the potential of XPO5 as a therapeutic target for TNBC. Given that the lncRNA SNHG4/XPO5 axis promotes the progression of TNBC, we will subsequently explore the potential of targeting the lncRNA SNHG4/XPO5 axis with drugs in the treatment of TNBC. Research indicates that treatment with oxaliplatin leads to downregulation of lncRNA SNHG4 expression in cells (38). After treatment with the ferroptosis inducer Erastin, lncRNA SNHG4 levels decrease in CRC cells (39). Drug sensitivity tests have identified chemotherapeutic agents that are negatively correlated with XPO5 expression, such as MG-132, paclitaxel, and WH-4-023 (40). This provides a new perspective for the clinical treatment of TNBC.

In conclusion, this study has preliminarily demonstrated that exosomes derived from the serum of TNBC patients promote the proliferation and migration of TNBC tumor cells, findings that were also validated in a nude mouse model. In-depth mechanistic studies revealed that the highly expressed lncRNA SNHG4 exerts its oncogenic effects by activating the XPO5-mediated pathway, thereby regulating TNBC cell proliferation and migration. These results not only enhance the understanding of the molecular mechanisms underlying TNBC progression but also suggest that exosomal lncRNA SNHG4 and XPO5 may serve as potential biomarkers for TNBC diagnosis and prognosis. Targeting the lncRNA SNHG4/XPO5 axis could offer a novel therapeutic strategy for TNBC.

## Data Availability

The data presented in the study are deposited in the Gene Expression Omnibus (GEO) (https://www.ncbi.nlm.nih.gov/geo/query/acc.cgi?acc=GSE300559) repository, accession number GSE300559.
