# Supplementary material for: Tumor-derived exosomal lncRNA SNHG4 promotes triple-negative breast cancer progression by targeting XPO5
Source: Front Oncol. 2025 Jun 27;15:1593827. doi: 10.3389/fonc.2025.1593827 (PMC12245919; doi:10.3389/fonc.2025.1593827)
Supplement: Supplementary file 1 [file DataSheet1.docx]

**Tumor-derived exosomal lncRNA SNHG4 promotes triple-negative breast cancer progression by targeting XPO5**

**Zhi-Wen Wang. et.al**

**Figure S1**


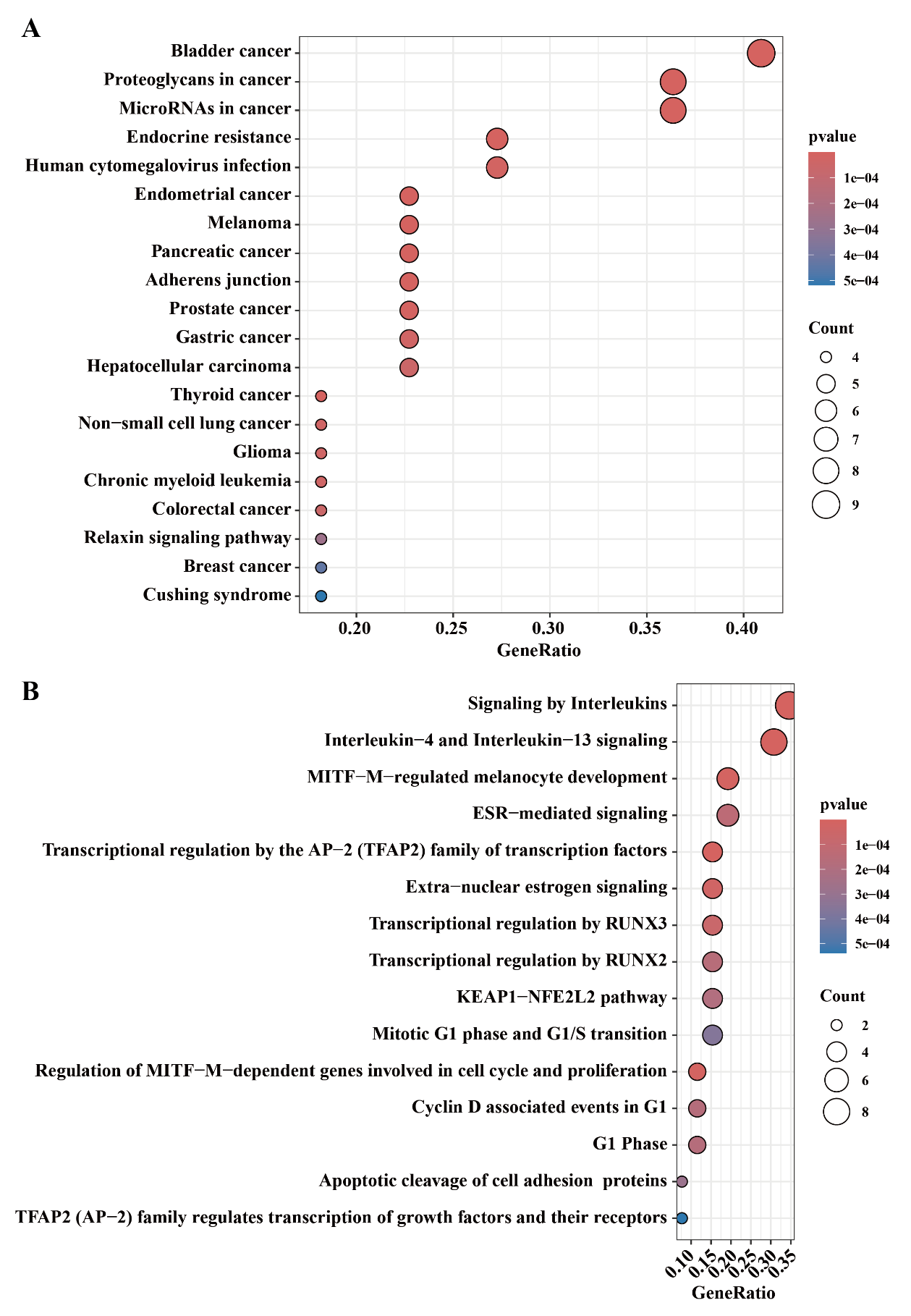


**Figure S1. KEGG and reactome pathway enrichment analysis of differentially expressed genes.** A. KEGG pathway enrichment analysis of differentially expressed genes from RNA sequencing. B. Reactome pathway enrichment analysis of differentially expressed genes from RNA sequencing.

**Figure S2**

**
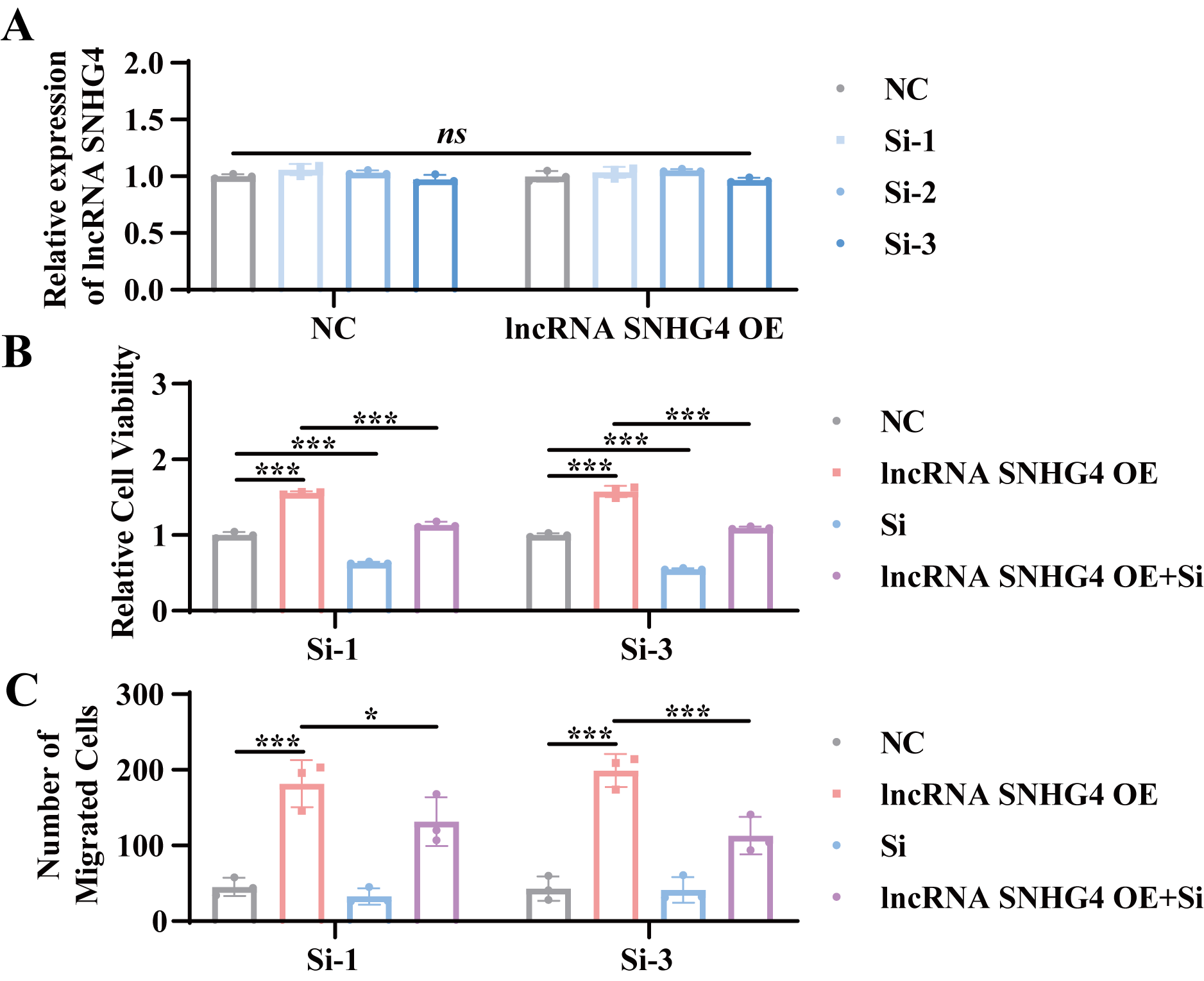
**

**Figure S2. LncRNA SNHG4 regulates tumor cell proliferation and migration through XPO5 in TNBC.** A. QRT-PCR analysis to detect relative XPO5 expression levels in stable lncRNA SNHG4-overexpressing and wild-type MDA-MB-231 cells 72 hours after transfection with NC or XPO5 siRNA. B. CCK-8 assay to assess relative cell viability in stable lncRNA SNHG4-overexpressing and wild-type MDA-MB-231 cells 72 hours after transfection with negative control (NC) or XPO5 siRNA. C. Transwell migration assay to evaluate relative cell migration ability in stable lncRNA SNHG4-overexpressing and wild-type MDA-MB-231 cells 72 hours after transfection with NC or XPO5 siRNA. Data are presented as Mean ± SD. Statistical significance was determined as follows: **p* < 0.05, ***p* < 0.01, ****p* < 0.001, *ns* > 0.05.
